# Prolonged Treatments With Antiresorptive Agents and PTH Have Different Effects on Bone Strength and the Degree of Mineralization in Old Estrogen-Deficient Osteoporotic Rats

**DOI:** 10.1359/jbmr.81005

**Published:** 2008-10-13

**Authors:** Zhiqiang Cheng, Wei Yao, Elizabeth A Zimmermann, Cheryl Busse, Robert O Ritchie, Nancy E Lane

**Affiliations:** 1 Department of Medicine, Aging Center, UC Davis Medical Center Sacramento, California, USA; 3 Materials Sciences Division, Lawrence Berkeley National Laboratory, and Department of Materials Science and Engineering, University of California Berkeley, California, USA

**Keywords:** PTH(1-34), intravenous bisphosphonates, bone mineralization, compression and bending strengths, bone mineral homogeneity

## Abstract

Current approved medical treatments for osteoporosis reduce fracture risk to a greater degree than predicted from change in BMD in women with postmenopausal osteoporosis. We hypothesize that bone active agents improve bone strength in osteoporotic bone by altering different material properties of the bone. Eighteen-month-old female Fischer rats were ovariectomized (OVX) or sham-operated and left untreated for 60 days to induce osteopenia before they were treated with single doses of either risedronate (500 μg/kg, IV), zoledronic acid (100 μg/kg, IV), raloxifene (2 mg/kg, PO, three times per week), hPTH(1-34) (25 μg/kg, SC, three times per week), or vehicle (NS; 1 ml/kg, three times per week). Groups of animals were killed after days 60 and 180 of treatment, and either the proximal tibial metaphysis or lumbar vertebral body were studied. Bone volume and architecture were assessed by μCT and histomorphometry. Measurements of bone quality included the degree of bone mineralization (DBM), localized elastic modulus, bone turnover by histomorphometry, compression testing of the LVB, and three-point bending testing of the femur. The trabecular bone volume, DBM, elastic modulus, and compressive bone strength were all significantly lower at day 60 post-OVX (pretreatment, day 0 study) than at baseline. After 60 days of all of the bone active treatments, bone mass and material measurements agent were restored. However, after 180 days of treatment, the OVX + PTH group further increased BV/TV (+30% from day 60, *p* < 0.05 within group and between groups). In addition, after 180 days of treatment, there was more highly mineralized cortical and trabecular bone and increased cortical bone size and whole bone strength in OVX + PTH compared with other OVX + antiresorptives. Treatment of estrogen-deficient aged rats with either antiresorptive agents or PTH rapidly improved many aspects of bone quality including microarchitecture, bone mineralization, turnover, and bone strength. However, prolonged treatment for 180 days with PTH resulted in additional gains in bone quality and bone strength, suggesting that the maximal gains in bone strength in cortical and trabecular bone sites may require a longer treatment period with PTH.

## INTRODUCTION

Osteoporosis is a disease that results from the deterioration of the material properties of such that the bone fractures with very little stress. Osteoporosis is common in elderly postmenopausal women, and statistically, nearly 50% of white women >50 yr of age will have an osteoporotic fracture in their lifetime.(1) Estrogen deficiency and aging-associated osteoporosis is referred to as a disease of bone remodeling, as increased bone turnover results in a reduction in bone mass, bone structure, and localized intrinsic properties of the bone including the degree of mineralization and bone strength. Currently, two types of medications are approved for the treatment of osteoporosis. These medications include antiresorptive agents that reduce bone turnover and prolong the secondary mineralization phase of bone, which results in a rapid improvement in bone strength because vertebral fracture risk reduction is observed within 6 mo of initiating the therapy.(2,3) Bisphosphonates in general increase lumbar spine BMD ∼3–4% after 1–2 yr of treatment.(4,5)

PTH(1-34) is an anabolic agent; hPTH(1-34) has also been approved for the treatment of osteoporosis and is effective in improving bone strength and reducing vertebral and nonvertebral fracture risk in postmenopausal women.(6) Daily injections of hPTH(1-34) result in a rapid increase in new bone formation followed by coupled bone remodeling for the duration of the therapy.(7) Trabecular bone is the primary surface on which PTH initially changes with increases in double labeled surface and thickness of the trabeculae. Treatment of postmenopausal women with PTH injections for 18 mo results in nearly 65% reduction in new vertebral fractures and a gain in lumbar spine BMD of nearly 9%.(8) The improved bone strength after PTH treatment is not associated with the gain in bone mass or the magnitude of the bone turnover changes.(8,9) Also, because the initial effects of PTH are directed to the trabecular bone surface, less is known about the effect of PTH on cortical bone. Anatomic sites with a high percentage of cortical bone initially lose bone mass with PTH treatment as a result of intracortical remodeling.(10,11) However, PTH stimulates bone formation more efficiently at active remodeling sites, and in aged female rats, estrogen deficiency increases remodeling on the endocortical bone surface.(12) Cortical bone has a slower remodeling rate than trabecular bone so any changes anticipated from PTH on cortical bone would tend to be observed after sites rich in trabecular bone, such as the lumbar spine.(13) The association of cortical bone mass changes and bone strength with PTH has not been carefully evaluated.

To further evaluate the changes in the intrinsic properties of bone that are influenced by estrogen deficiency, aging, and osteoporosis treatment agents, investigators have initiated studies that attempt to quantify the degree of bone mineralization and the distribution of the mineral across the bone surface, the crystal size and orientation, and the material properties of the bone surface by elastic modulus mapping.(14–19) We hypothesized that aged osteopenic female rats treated with either antiresorptive agents or an anabolic agent, PTH, would gain bone mass and have changes in the material properties of bone such that bone strength would improve. We further hypothesized that prolonged treatment with these agents would provide additional changes to the material properties of the bone that might result in greater bone strength. We found that short-term treatment with both agents returned the bone mass and bone strength to baseline levels. However, prolonged treatment with PTH resulted in additional gains in both trabecular bone volume and mineralization, in cortical bone mass, and in whole bone and compressive strength compared with sham-operated animals and animals treated with antiresorptive agents. These observations suggest that prolonged treatment of osteoporotic women with PTH may have additional advantages in bone strength through changes that occur in the intrinsic properties of the bone matrix.

## MATERIALS AND METHODS

### Animals and experimental procedures

Female Fischer 344 rats were purchased from NIA (Bethesda, MD, USA) and maintained on commercial rodent chow (22/5 Rodent Diet; Teklad, Madison, WI, USA) available ad libitum with 0.95% calcium and 0.67% phosphate in a room that was maintained at 21°C with a 12-h light/dark cycle. At 18 mo of age, the rats were randomized by body weight into 15 experimental groups, and animals were subjected to either sham surgery or ovariectomy (OVX) (Table 1). The animals in groups 2–15 were left untreated for 60 days to allow for the development of trabecular osteopenia. At day 60 after OVX (day 0 of the intervention study), groups 2 (pretreatment Sham control, *n* = 6) and 3 (pretreatment OVX control, *n* = 6) were killed. The other remaining animals were treated with either vehicle (normal saline, SC, three times per week), risedronate (Ris, 500 μg/kg, single IV injection at day 0), zoledronic acid (Zol, 100 μg/kg, single IV injection at day 0), raloxifene (Ral, 2 mg/kg, oral gauge, three times per week, days 0–180), or hPTH(1-34) (25 μg/kg, SC, three times per week, day 180; Table 1). The medication dosages used in this experiment were derived from publications by the manufacturers that have been shown in osteopenic animals models to be effective and similar in concentration to clinical doses.(20–23) Twenty-four-hour urine samples were collected every 60 days and were stored at –80°C until they were used to assessment of biochemical markers of bone turnover. Xylenol orange (90 mg/kg) was given to all rats except animals killed at baseline (day −60). All other animals received calcein (10 mg/kg) followed by alizarin red (20 mg/kg) subcutaneously at 14 and 4 days before death to assess bone formation surface. At the time of death, the right tibias were placed in 10% phosphate-buffered formalin for 24 h and transferred to 70% ethanol for high-resolution CT (μCT), bone histomorphometry, and scanning probe microscopy (SPM). The fourth lumbar vertebral bodies (LVB) were scanned by μCT, and the fourth LVB and the left femurs were used for biomechanical testing.

**Table 1:** Study Groups and Experimental Design

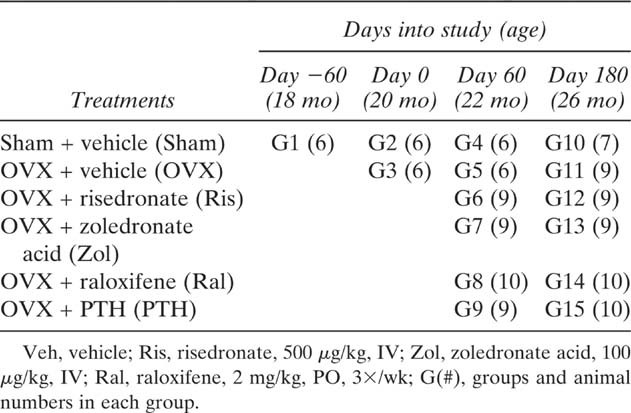

All animals were treated according to the USDA animal care guidelines with the approval of the UC Davis Committee on Animal Research.

### Biochemical markers of bone turnover

Urinary levels of free deoxypyridinoline (DPD) cross-links and osteocalcin were measured using an enzyme-linked immunoassay (EIA) kit (DPD; Quidel; osteocalcin, Biomedical Technologies, Stoughton, MA, USA). The manufacturer's protocols were followed, and all samples were assayed in duplicate. A standard curve was generated from each kit, and the absolute concentrations were extrapolated from the standard curve. The CVs for interassay and intra-assay measurements were <10% for all assays and are similar to the manufacturer's references. The DPD values were corrected for Cr concentrations and were expressed as nmol DPD/mmol Cr.(24,25)

### μCT measurements of the trabecular bone microarchitecture and degree of bone mineralization

The right proximal tibias (PTM) and the fourth lumbar vertebral bodies (LVBs) were scanned with μCT (VivaCT 40; Scanco Medical, Bassersdorf, Switzerland) at an energy level of 70 kev and intensity of 85 μA with an isotropic resolution of 10.5 μm in all three spatial dimensions. Scanning for the PTM was initiated proximally at the level of growth plate and extended distally for 210 slices. Evaluations were performed on 100 slices beginning from ∼0.2 mm distal to the growth plate. The entire LVB was scanned, and the trabecular bone within the cranial and caudal growth plates and the cortex was evaluated. The grayscale images were segmented using a constrained 3D Gaussian filter (sigma = 0.8, support = 1.0, a fixed threshold of 240) to extract the structure of mineralized tissue. Because the scanner is self-calibrating before every scan, a fixed threshold provides the most reproducible result.

Trabecular bone volume (BV) was calculated using tetrahedrons corresponding to the enclosed volume of the triangulated surface. Total volume was the volume of the sample that was examined. A normalized index, BV/TV, was used to compare samples of varying bone size. To minimize the partial volume effects, two surface voxels were discarded from every trabecula such that thick trabeculae (with 6 or more pixels) would be counted. The methods used for calculating connectivity density, trabecular number, and trabecular thickness have been described previously.(24,26,27) In addition, the attenuation coefficient (cm^−1^) of each pixel was calculated, and the histogram of bone material concentration was derived for each animal to calculate the mean degree of bone mineralization (DBM) and mineralization distribution curve. The g HA/cm^3^ values were standardized with a manufacturer-supplied phantom of five different HA densities embedded in soft-tissue equivalent resin. Beam hardening effects were corrected in the reconstruction process with a correction curve adapted to individual bone scans, as supplied by the manufacturer. The degree of mineralization was calculated from the whole bone sample region used for architectural analyses.(15,16) The average mineralization (Ave-MIN) was defined average mineralization of the whole trabecular bone sample. Peak mineralization (Peak-MIN) was defined as the most frequent encountered mineralization obtained from the histogram of mineralization.(16) Low-MIN (%) was defined as the percentage of bone volume with mineral <0.8 g/cm^3^, medium-MIN (%) was defined as the percentage of bone volume with mineral 0.8–1.2 g/cm^3^, and high-MIN (%) was defined as the percentage of bone volume with mineral >1.2 g/cm^3^.

### Measurement of elastic modulus by scanning probe microscopy

**FIG. 5:**
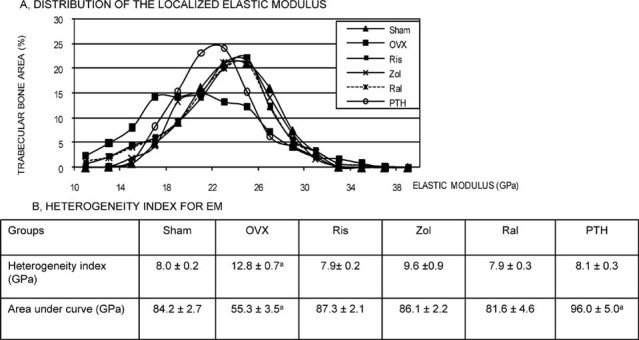
Localized elastic modulus of the PTM trabecular surface measured at day 180. OVX + vehicle induced a significant reduction in elastic modulus and increased the heterogeneity. OVX + Ris, Zol, or Ral and sham had similar elastic modulus, which had a greater percentage of high elastic modulus compared with OVX + PTH. The heterogeneity index of antiresorptive treatments and PTH was similar to that of sham. However, PTH had the highest overall Elastic modulus compared with all the other groups at 180 days. ^a^*p* < 0.05 from all the other groups.

After taking three 4-μm and one 8-μm sections for bone histomorphometry, the remaining methylmethacrylate-embedded proximal tibia blocks were further polished with different diamond pastes starting from 10 μm down to 0.1 μm diameter to obtain smooth surfaces. We used a force modulation technique called dynamic stiffness mapping (DSM) to quantitatively map dynamic nanomechanical properties with nanometer scale resolution.(28) Modulus maps in the form of scanning probe microscopy (SPM) images were acquired using the direct-force modulation operating mode of a TriboScope nanoindenter (Hysitron, Minneapolis, MN, USA) mounted on a Multimode atomic force microscope (AFM) controlled by NanoScope IIIa electronics (Veeco, Santa Barbara, CA, USA). In this experiment, the conventional AFM head was replaced by an electrostatic operated transducer for simultaneous topographic and elastic modulus imaging. The topographic contact mode was used first to locate the area of interest, usually 50 × 50 μm^2^. Subsequently, the same area was scanned in force modulation mode to record the elastic modulus map. The amplitude of the modulated electrostatic force was set to 0.5–1.0 μN to maintain good signal-to-noise ratio but was maintained sufficiently small to prevent plastic deformation of the sample. Voigt and Hertzian models were used to extract the elastic modulus map of 256 × 256 pixels from the amplitude and phase of displacements at each pixel.(19) One representative PTM was studied per group, with six different areas (images) on each sample, and each area (or image) had 65,536 pixels. We obtained statistics on 393,216 points for each sample. The localized SPM distribution was plotted as the percentage of elastic modulus over the trabecular area studied (Fig. 5A). A heterogeneity index of the surface elastic modulus was adapted from the methods described by Biovin and colleagues.(14,29) It was defined as the “full width at half maximum” of the individual elastic modulus mapping distribution curve across the trabecular surface. The area underneath the curve was also calculated to determine whether there were differences in the elastic modulus between the experimental groups (Fig. 5B).

We assessed lacunar size and volume by two different methods. The sizes of lacunae were estimated from the polished sections of trabecular bone using the same diamond tip used for the elastic modulus mapping. The lacunae with the largest areas were located and assumed to be the closest approximation to the mid-cross-section of a 3D ellipsoidal lacuna. These methods are similar to those reported in our previous work.(19)

### Bone histomorphometry measurements of the tibial trabecular and cortical bone

The right proximal tibial metaphyses (PTM) and the shaft of the right tibia were dehydrated in ethanol and embedded undecalcified in methylmethacrylate. The PTMs were sectioned longitudinally with a Leica/Jung 2255 microtome in 5- and 8-μm-thick sections. The 5-μm sections were stained with tetrachrome stain for collection of bone mass and architecture data with the light microscope, whereas the 8-μm sections were left unstained for measurement of fluorochrome-based indices. The tibial shafts were cut into 38-μm sections using a precision diamond wire saw (Well diamond Wire Saw, Norcross, GA, USA). The first section proximal to the tibial-fibula joint was selected for evaluation. Bone histomorphometry was performed using a semiautomatic image analysis Bioquant system (Bioquant Image Analysis, Nashville, TN, USA) linked to a microscope equipped with transmitted and fluorescent light.

Bone histomorphometric analyses were performed in the secondary spongiosa of the right proximal tibias that included trabecular area between 0.1 and 3 mm proximal to the growth plate and within the cortex. Bone turnover measurements included single- (sL.Pm) and double-labeled perimeter (dL.Pm), interlabel width (Ir.L.Wi), and osteoclast surface. These indices were used to calculate mineralizing surface (MS/BS), mineral apposition rate (MAR), bone formation rate (BFR/BS), and percentage of osteoclast surface (Oc.S).(24,25) Cortical bone measurements included total cortical cross-sectional area (mm^2^), marrow area (%), and cortical bone thickness (μm).(19,24,25),(30–32)

### Biomechanical testing

The mechanical properties of the bone, specifically the maximum compression and bending strengths, were determined using both lumbar compression tests on vertebrae and three-point bending on un-notched femurs.

Biomechanical testing of the fourth lumbar vertebrae (LVB) was performed using an axial compression test in ambient air. The relevant cross-sectional dimensions and the height of the specimens were measured using an optical microscope with a 0.5-μm resolution (STM-UM Measuring Microscope; Olympus American, Melville, NY, USA), after which they were subjected to unconfined compression tests along the long axis of the lumbar vertebra with a cross-head displacement rate of 0.01 mm/s. This test involved loading the samples to failure while continuously recording the corresponding loads and displacements. Maximum load and the cross-sectional dimensions were measured and used to determine the maximum compression strength.(24,25)

The femora were received in 70% ethanol solution. The ends of the femora were removed with a low-speed saw, and the femora were placed in HBSS for at least 24 h before testing. To determine the bending strength of the cortical bone, the femora were placed in an articulating, three-point bending fixture (with a lower-support loading span of *L* = 15 mm). Specimens were placed onto the lower support such that the bending axis corresponded with the sagittal plane. The upper loading point was brought in contact with the anterior side of the femur, and the specimen was loaded to failure in 37°C HBSS at a displacement rate of 0.01 mm/s using a servo-hydraulic materials testing system (Model 831; MTS, Eden Prairie, MN, USA). The applied force was measured with a calibrated load cell (Sensotec Model 31), whereas the support travel was measured with the system linear variable differential transformer. After testing, a two-point average of the diameter (anterior-posterior and medial-lateral) and a six-point average of the thickness were measured at the fracture site of each femur using digital calipers. Using the average dimensions, the area moment of inertia was calculated by approximating the cross-section as annular. The maximum bending strength, σ_max_, and the macroscopic elastic modulus, *E*, were measured from the standard beam bending equations in terms of the span length, *L,* the maximum load, *P*:


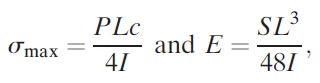


where *c* is the distance on the cross section from the point of maximum tension to the neutral axis, *I* is the moment of inertia of the circular cross section, and *S* is the slope of the most linear region of the force versus displacement data.(11,33,34)

### Statistical analysis

The group means and SDs were calculated for all outcome variables. The nonparametric Kruskal-Wallis test was used to determine differences at the same time point with posthoc comparisons performed by Tukey's test between groups at the same time point. (Version 12; SPSS, Chicago, IL, USA). Differences were considered significant at *p* < 0.05.

## RESULTS

### Validation of the model

#### Estrogen deficiency induced trabecular bone loss and increased bone turnover in the aged female rats at day 60 postovariectomy

**FIG. 1:**
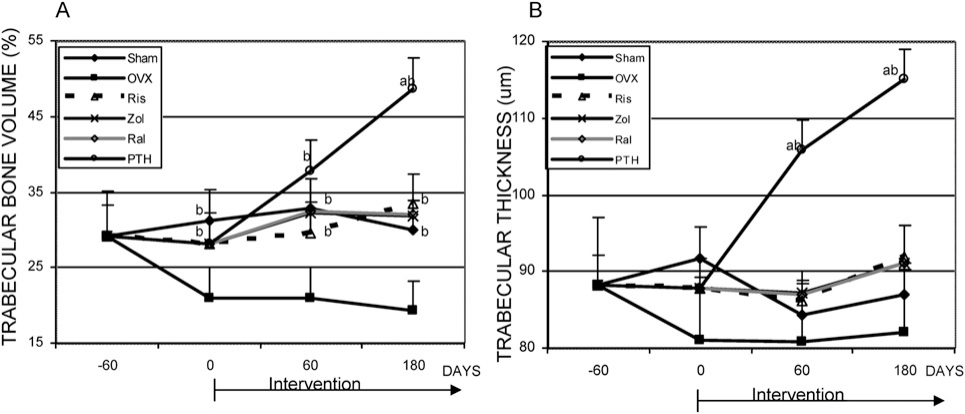
Proximal tibial trabecular bone microarchitecture changes measured by μCT. Mean values and SD for (A) trabecular bone volume and (B) trabecular bone thickness from the right proximal tibial metaphyses obtained from groups of sham-operated (Sham) or OVX animals treated with vehicle (OVX), risedronate (Ris), zoledronic acid (Zol), raloxifene (Ral), and PTH(1-34) from days 0 to 180. ^a^*p* < 0.05 vs. sham-operated animals at the same time point; ^b^*p* < 0.05 vs. OVX + vehicle-treated animals at the same time point.

**FIG. 2:**
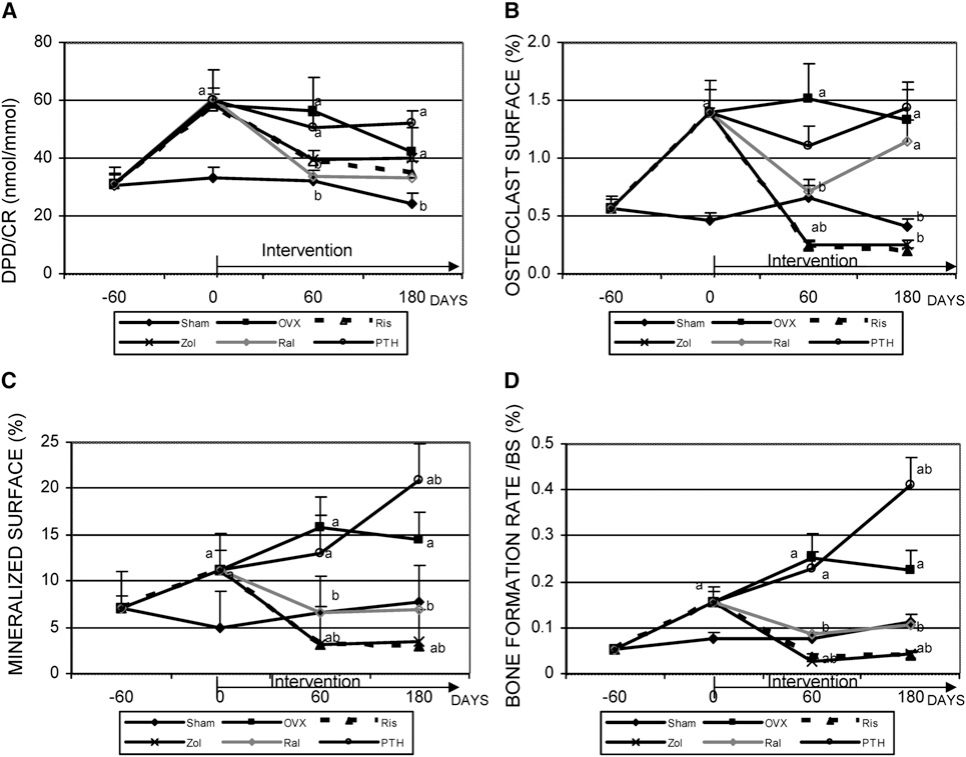
Serum biochemical and surface based assessments of bone turnover. Mean values and SD for (A) urine DPD (nmol) cross-links and creatinine (Cr, mmol) measured by ELISA; (B) percent osteoclast surface (%); (C) percent mineralizing surface (%); and (D) percent surface-based bone formation rate (μm/d). (B-D) Measured from bone histomorphometry obtained from the proximal tibial metaphysis for groups of sham-operated (Sham) or OVX animals treated with vehicle (OVX), risedronate (Ris), zoledronic acid (Zol), raloxifene (Ral), and PTH(1-34) (PTH) from days 0 to 180. ^a^*p* < 0.05 vs. sham-operated animals at the same time point; ^b^*p* < 0.05 vs. OVX + vehicle-treated animals at the same time point.

Compared with the baseline group (day 60), there was no difference in BV/TV or bone turnover in the sham-operated group at day 60 (day 0 of intervention study). However, after 60 days of estrogen deficiency, ovariectomized animals lost 33% trabecular bone mass at the proximal tibia (Fig. 1) and 24% at the lumbar vertebrae compared with the sham-operated animals at day 60 (*p* < 0.05). The majority of estrogen-deficient trabecular bone loss occurred during the first 60 days postovariectomy. At day 60, postovariectomy bone resorption, measured by urine DPD cross-links (DPD/Cr), at the osteoclast surface increased 2- to 3-fold. Also at day 60, postovariectomy, bone formation measured by trabecular mineralizing surface and surface-based bone formation rate increased 1- to 2-fold (Fig. 2; *p* < 0.05 compared with sham at the same time point).

### Intervention study (days 0–180)

#### Single intravenous doses of risedronate and zoledronic acid and oral raloxifene restored trabecular bone mass, whereas PTH(1-34) increased trabecular bone mass in aged estrogen-deficient rats

We used μCT to define the effects of single intravenous doses of Ris and Zol, Ral, and PTH(1-34) treatments on bone microarchitecture compared with the vehicle-treated OVX animals. All antiresorptive agent, Ris, Zol, and Ral, treatments of OVX rats significantly increased bone volume (BV/TV) compared with vehicle-treated OVX animals by day 60 of treatment, were 42%, 54%, and 55% greater than OVX + vehicle-treated animals at day 60 (*p* < 0.05; Fig. 1), respectively, and were similar to the sham-operated animals. At day 180, Ris, Zol, and Ral treatments of OVX animals further increased BV/TV to 72%, 64%, and 65% greater than the OVX + vehicle-treated animals at day 180, respectively (*p* < 0.05; Fig. 1), and were all nearly 10% higher than the sham-operated group (*p* < 0.05; Fig. 1). In contrast, after 60 days of treatment, PTH(1-34) BV/TV and trabecular thickness (Tb.Th) increased by 80% and 31%, respectively, compared with the OVX + vehicle-treated animals (*p* < 0.05; Fig. 1). At day 180, in PTH-treated animals, BV/TV and Tb.Th were 150% and 40% greater than OVX + vehicle-treated animals and were 63% and 32% greater than the sham-operated group (*p* < 0.05; Fig. 1), respectively. Also, PTH treatment resulted in BV/TV that was significantly greater than all other antiresorptive + OVX treatment groups at day 180.

**FIG. 3:**
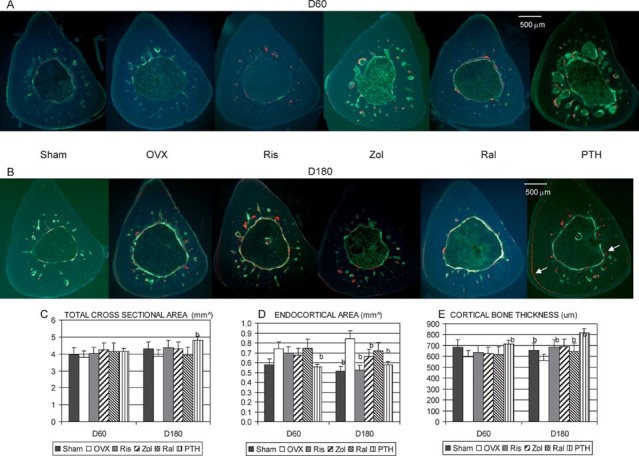
Tibial shaft cortical bone histomorphometry. Unstained cross-sections of the midtibial shafts from (A) day 60 and (B) day 180 animals treated with vehicle, vehicle (OVX), risedronate (Ris), zoledronic acid (Zol), raloxifene (Ral), and PTH(1-34) (PTH). Note the enlarged marrow cavities in OVX and Ral groups at day 180 and the markedly reduced marrow cavities in Ris, Zol, and PTH groups at day 180. At day 60, Zol and PTH had higher intracortical remodeling (labeled osteon) in the inner third of the cortex. PTH increased total cross-sectional area and decreased endocortical area at day 180 (C). (D) Treatment with PTH increased the cortical thickness at both days 60 and 180, whereas with Ris and Zol increased cortical thickness at day 180 (E).

The PTH treatment group had higher total-cross sectional area than the rest of the groups at day 180 (Figs. 3A and 3C). Endocortical area was higher in the OVX + vehicle and OVX + Ral groups than the other groups at day 180 (Fig. 3D). Cortical bone thickness was 20% higher in the 60-day OVX + PTH(1-34) group and also in the OVX + Ris (+15%), OVX + Zol (+22%), and OVX + PTH(1-34) (+37%) groups at day 180 compared with the OVX + vehicle group (*p* < 0.05; Fig. 3E).

#### Antiresorptive treatments decreased, whereas PTH(1-34) increased, bone turnover compared with the ovariectomized animals

Compared with the OVX + vehicle-treated animals, OVX animals treated with single intravenous doses of Ris or Zol and oral Ral had nearly a 50% reduction in urine DPD/Cr and osteoclast surface so that the levels were similar to the sham-operated levels at both day 60 and day 180 of the treatment period (*p* < 0.05). OVX + PTH(1-34) treatment had a 59% increase in urine DPD/Cr levels at day 60 and was similar to the OVX + vehicle group but further increased at day 180 to 158% greater than the sham-operated group (*p* < 0.05; Fig. 2). The OVX + PTH group had similar OcS/B.S values as the OVX + vehicle group at day 60 and day 180, but OcS/B.S. was 141% greater at day 60 and 251% greater at day 180 compared with the sham-operated group (*p* < 0.05; Fig. 2).

Bone formation rates measured by bone histomorphometric analysis of trabecular bone in the proximal tibial metaphysis indicated that OVX rats treated with either Zol or Ral had similar mineralized surfaces and bone formation rates/BS at days 60–180 compared with the sham-operated rats. In contrast, OVX + Ris animals had significantly decreased bone formation parameters (*p* < 0.05) compared with sham and OVX + vehicle-treated groups (*p* < 0.05; Fig. 2). Treatment with OVX + PTH(1-34) significantly increased mineralizing surface and bone formation rate/BS by nearly 100% at day 60 compared with the sham-operated animals and were similar to OVX + vehicle animals. At day 180, for the OVX + PTH group, these parameters were increased by 43% and 86% compared with the vehicle-treated animals and 150% greater than the sham-operated animals (*p* < 0.05; Fig. 2). Interestingly, double-labeled osteocyte surface was assessed in the trabecular bone sections and was similar at day 60 for OVX + vehicle and sham-operated animals (two of total trabecular region) but was significantly higher in the OVX + PTH group (eight of total trabecular region) (data not shown). However, by day 180, there was no double-labeled osteocyte surface observed in any experimental group.

#### Antiresorptive treatment restored the lost trabecular degree of bone materialization after OVX, whereas PTH(1-34) increased the degree of bone mineralization

**FIG. 4:**
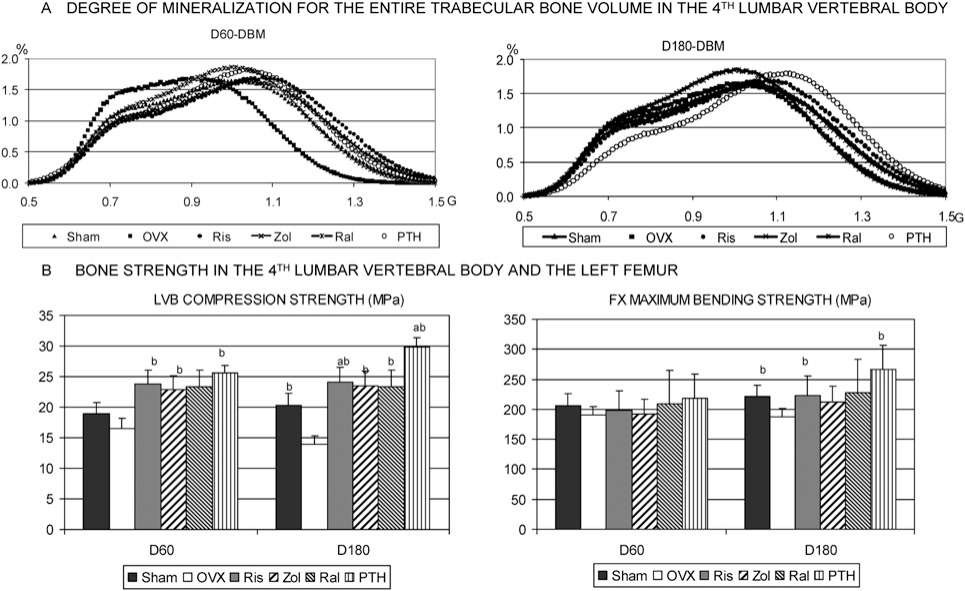
(A) Degree of bone mineralization (DBM) and mineral distribution generated by high-resolution μCT for trabecular bone of the fourth lumbar vertebral bodies at days 60 and 180. The distribution of mineralization shifted to the left with OVX at day 60, with lower values of DBM. All the treatment groups had similar trabecular mineral distribution curves as the sham controls at day 60. At day 180, Ris, Zol, and PTH treatment shifted the mineral distribution to the right, with more bone of higher mineral concentration. (B) Lumbar vertebral compression strength and femoral maximum bending strength at days 60 and 180. At day 60, antiresorptive treatments and PTH had similar lumbar compression strength that was significantly higher than the OVX group. At day 180, PTH further increased lumbar compression strength, which was higher than all the other groups. Femoral bending strengths were increased with Ris and PTH treatments compared with the OVX at day 180. ^a^*p* < 0.05 vs. sham at the same time point; ^b^*p* < 0.05 vs. OVX at the same time point; ^c^*p* < 0.05 vs. the antiresorptive groups.

We next determined how single intravenous doses of either Ris or Zol and long-term oral Ral treatments in old OVX osteopenic rats affected the degree of bone mineralization. High-resolution μCT of the LVB and PTM was used to determine the degree and DBM. Because trends for DBM changes with all interventions were similar for the two trabecular anatomic sites (PTM and LVB), we describe here the results for the LVB. The Ave-MIN and Peak-MIN in OVX + vehicle-treated animals did not differ significantly from the sham-operated group at days 60 and 180 (Fig. 4A; Table 2). Compared with all other groups, the distribution of the DBM from OVX + vehicle-treated animals was shifted to the left, especially at day 60, because of a greater volume of bone with a higher percentage of lower mineral values (14% and 8%, respectively, higher than sham at days 60 and 180; Fig. 4A; Table 2). OVX + Ris or Zol groups had similar Ave-MIN, Peak-MIN, and DBM distribution as the sham-operated animals at day 60 (Fig. 4A; Table 2). At day 180, OVX + Ris or Zol groups had higher Ave-MIN and Peak-MIN and a higher percentage of high-MIN than the OVX + vehicle group (Fig. 4A; Table 2). The changes with OVX + Ral were between the OVX + vehicle and sham + vehicle groups. OVX + PTH(1-34)–treated animals had Ave-MIN, Peak-MIN, and mineralization distribution at day 60 that was similar to the OVX + vehicle-treated animals. However, Ave and Peak-MIN were ∼7% and 9% higher by day 180 compared with the OVX + vehicle group (Fig. 4A; Table 2), which was similar to the OVX + Ris or Zol groups. The distribution of mineralization at day 180 in the OVX + PTH(1-34) group was shifted to the right, because of a greater volume of bone with higher mineral concentration, which was similar to the OVX + Ris or Zol groups.

**Table 2:** Degree of Bone Mineralization of the Vertebral Trabecular Bone

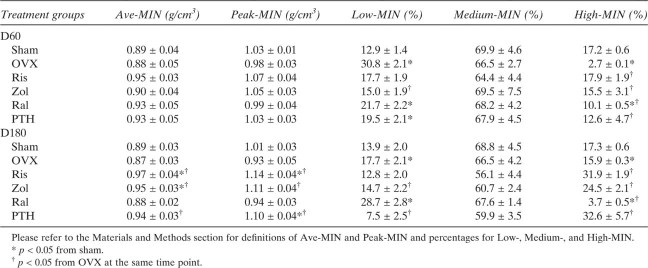

#### Antiresorptive treatment restored, whereas PTH(1-34) increased, localized and whole bone strength in osteopenic ovariectomized rats

Mechanical compression testing of the fourth lumbar vertebrae indicated that OVX + vehicle resulted in a significant reduction in compression strength (Fig. 4B) and stiffness (data not shown) compared with those in the sham-operated groups at days 60 and 180 (*p* < 0.05; Fig. 5). The maximum compression strength was ∼70% higher in the Ris, Zol, and Ral + OVX treatment groups compared with the OVX + vehicle-treated animals at day 180 (*p* < 0.05), which was similar to the sham-operated animals. The OVX + PTH group had 114% higher compression strength compared with the OVX + vehicle-treated group at day 180 (*p* < 0.05) and were significantly higher than the sham-operated group (*p* < 0.05). It should be noted that the compression strength of the vertebrae is significantly lower than the bending strength of the femurs; this is most likely associated with the much higher fraction of trabecular bone in the vertebrae.

With respect to the maximum bending strength of the cortical bone, three-point bending testing of the femurs indicated that OVX + vehicle resulted in a significant reduction in strength compared with those in sham-operated groups at day 180 (*p* < 0.05; Fig. 4B). The bending strength was ∼18% higher in the Ris + OVX treatment groups compared with the OVX + vehicle-treated animals at day 180 (*p* < 0.05), which was similar to the sham-operated animals. OVX + PTH groups had 42% higher bending strength compared with the OVX + vehicle-treated group at day 180 (*p* < 0.05) but was not significantly higher than the sham-operated group.

Localized elastic modulus values of the PTM trabecular surface measured at day 180 showed that OVX + vehicle induced a significant reduction in elastic modulus (stiffness) together with increased the heterogeneity (Fig. 5). The OVX + Ris, Zol, or Ral and sham groups all displayed similar elastic moduli and had a greater percentage of high elastic modulus values compared with OVX + PTH. The heterogeneity index of antiresorptive treatments and PTH was similar to that of sham (Fig. 5). However, PTH had the highest overall elastic modulus compared with all other groups at 180 days (*p* < 0.05). In addition, osteocyte lacunae size, measured from the SPM, found that the average size of nearly 50 osteocyte per treatment group was lower in PTH-treated animals than OVX + vehicle and OVX+ antiresorptive agents (mean ± SD: 40.1 ± 7.5 μm^2^ versus OVX + vehicle [68.7 ± 15.5 μm^2^] versus OVX + antiresorptive agents [59 ± 10.7 μm^2^]).

## DISCUSSION

The determinants of bone fragility in osteoporosis and how currently approved treatments for osteoporosis augment bone strength independent of changes in BMD are still being defined.(35–37) Therefore, we evaluated the effects of antiresorptive agents and PTH on other properties of bone quality including bone microarchitecture, surface-based and biochemical markers of turnover, mineralization, crystal size, and whole bone material properties in aged estrogen deficiency OVX rats for 60 and 180 days. We found that treatment of osteopenic aged rats with either antiresorptive agents or PTH restored bone mass, total trabecular and cortical mineralization, and strength after 60 days, with turnover reduced with antiresorptive agents and increased with PTH. However, from day 60 to day 180, the PTH-treated group had additional gains in bone mass, bone mineral concentration, and trabecular and cortical bone strength. Our data suggested that over time PTH treatment may be superior to antiresorptive agents and that it may have a greater influence on cortical bone strength.

In clinical medicine, antiresorptive agents and PTH are prescribed for individuals with low bone mass or osteoporotic fractures and both seem to reduce the risk of new incident vertebral fractures by a similar magnitude(4,5),(38–44); however, risedronate seems to have a more rapid action, with significant vertebral fracture reduction after 6 mo of therapy.(3) The bone strengthening properties of bisphosphonates result from a reduction in the initiation of bone remodeling. This allows for a prolongation of the secondary mineralization phase and more total bone mineral, a lower ratio of low to highly mineralized bone, and a more uniform distribution of mineral, which are all associated with improved bone strength.(14–16,29),(45–48) Our work and that of others have reported that the degree of mineralization in trabecular bone is a strong predictor of bone strength. The majority of preclinical studies based on short-term intervention studies of 2–3 mo report an increased degree of bone mineralization seems to be advantageous to bone strength. However, the clinical use of these medications is for many years. Although long-term use of oral bisphosphonates seems to maintain bone strength over 10 yr, it is not clear if the change in bone mass or degree of bone mineralization is associated with the maintenance of this improved bone strength.(49)

Another aspect of bone strength is the rate of bone remodeling, which results in the release of mineral and collagen from the extracellular matrix. Coupled remodeling causes a removal and replacement of both the mineral and unmineralized matrix. Estrogen deficiency uncouples remodeling with increased osteoclast and osteoblast maturation and activity. Biochemical markers of bone turnover that are correlated with the uncoupling of bone remodeling include TRACP 5b, urinary DPD, and serum osteocalcin. In this study, treatment with antiresorptive agents reduced remodeling rates and rapidly lowered biochemical markers of bone turnover. This was confirmed by surface-based histomorphometry after treatment initiation and was maintained throughout the treatment period. In contrast, treatment with PTH increased bone turnover with increased urine and serum markers of osteoclast maturation and activity, osteoblast activity, and turnover by histomorphometry. Reduction in bone turnover over time with antiresorptive agents inhibits the replacement of the bone matrix. However, PTH stimulates remodeling and replaces the matrix with divalent cross-links of collagen I, which is characteristic of younger bone, whereas older bone has more trivalent cross-links.(50,51) Bone biopsies from osteoporotic women treated with PTH and placebo for 18 mo had a lower ratio of trivalent to divalent cross-links.(51) Divalent cross-links are reported to decrease fracture load and offset yield load; however, trivalent cross-links enhance bone toughness.(52) Therefore, we hypothesize that antiresorptive agents may increase the concentration of trivalent cross-links in bone, thereby enhancing bone toughness, whereas PTH may improve bone strength by increasing divalent cross-links despite initially reducing matrix mineralization and mineral crystallinity. Additional studies that include multiple doses of both antiresorptive agents and PTH are needed to test this proposed hypothesis.

Because this experiment used aged female rats, we studied the surface-based turnover at both the trabecular and cortical bone sites and found evidence for both endocortical and Haversian remodeling in the cortical bone of these aged estrogen-deficient rats. It is generally believed that Haversian remodeling does not present in the rat species. However, it can be induced under circumstances such as after fatigue loading(33) or thyroparathyroidectomized(53)-induced hypocalcemia. In this study, the animals were >18 mo old when the study was initiated and nearly 30 mo at the termination of the study. Very few studies, if any, have conducted extensive histological analysis with this age of rat. Sixty days of PTH treatment elevated double-labeled surfaces on the trabecular, endocortical, and the peri-osteocyte lacunae surfaces in areas adjacent to the remodeling sites. The double-labeled bone around the osteocyte lacunae was present at both the 60- and 180-day time points. Other groups have reported young osteocytes have PTH receptors and can form nodules when exposed to PTH in vitro; Midura et al.(54,55) reported calcein labeling around osteocytes near the periosteum of rats treated with PTH. The significance of PTH's ability to stimulate bone formation around the osteocyte is not known. The size of the osteocyte lacunae of PTH-treated animals was significantly less than animals treated with antiresorptive agents, vehicle-treated OVX animals, and sham controls in this study. The osteocyte is responsible for sensing the loads of the bone through the fluid that flows through it. The extracellular matrix that surrounds the osteocyte senses the load and transmits it through SIBLING proteins to the osteocyte. Osteocyte lacunae size is hypothesized to be associated with how effective the bone is at absorbing shear force. Smaller osteocyte lacunae may be better able to absorb shear forces than larger lacunae. Inability of the osteocyte to absorb shear force can result in microcracks, possibly apoptosis, or signals sent to the surface of the bone that initiate remodeling.(56,57) In previous reports, we showed that increased osteocyte lacunae size was associated with a loss of mineral surrounding the osteocyte in glucocorticoid-treated mice(19) and reduced localized elastic modulus. Both acute and prolonged estrogen deficiency is reported to induce apoptosis of osteocytes in vivo,(58–60) and both estrogen and selective estrogen receptor modulators were able to inhibit this effect in vitro.(60,61) The OVX + vehicle-treated animals in this study were estrogen deficient for nearly 240 days, and this group had larger osteocyte lacunae size and increased Haversian remodeling in the cortex. Additional studies are needed to determine whether lacunae size is related to apoptosis of the osteocytes with estrogen deficiency and whether treatment with an antiresorptive agent or PTH can alter localized bone strength related to the changes in osteocyte and lacunae sizes.

After 60 days of PTH treatment, estrogen-deficient animals had improved bone mineralization of the trabecular bone from the lumbar vertebrae compared with the OVX + vehicle-treated group. However, by day 180, the PTH-treated animals had a higher percentage of trabecular bone with higher mineral that was similar to the animals treated with bisphosphonates. PTH initially increases bone turnover and cortical bone porosity,(9,11) and we found that PTH initially stimulated bone turnover, created a mineralization lag,(51,62) and increased cortical bone remodeling. However, at day 60, bone strength was similar in PTH- and bisphosphonate-treated animals, suggesting that high turnover in the presence of improved bone mass and microarchitecture was a more important determinant for bone strength; PTH treatment from days 60 to 180 resulted in nearly 30% greater bone mass with a shift toward more highly mineralized bone in the trabecular bone regions, filling in of the intracortical bone remodeling spaces, and an increase in cortical bone thickness. The prolonged treatment with PTH may have allowed for the new osteoid to completely mineralize. The further increase in bone mass and degree of mineralization further augmented bone strength in both trabecular and cortical bone sites.

Localized material properties of trabecular bone can provide additional information about overall bone quality. Such measurements were made using a modulus mapping technique, which permitted multiple measurements of the elastic modulus or stiffness across the trabecular surface of the bone. The elastic modulus that was measured is the so-called Young's modulus, which is a measure of the stiffness of the bone (it is the tensile or compressive stress needed for unit increase in elastic deformation). The scanning probe-based modulus mapping technique is particularly useful for providing an accurate means of assessing the stiffness of small entities such as trabeculae.

Previous experiments of glucocorticoid treatment and estrogen deficiency showed a reduced local elastic modulus at the trabecular bone surface and within the trabecular around the osteocyte lacunae; this reduced stiffness in glucocorticoid-treated mice bone was associated with reduced bone mineral to matrix ratio assessed by Raman spectroscopy.(19,34) In this study, 240 days of estrogen deficiency was associated with a higher percentage of trabecular surface with lower elastic modulus compared with antiresorptives and PTH. PTH treatment produced an overall increase in surface elastic modulus with similar distribution of elastic modulus, as observed with the sham-operated and antiresorptive treatment groups. The extent in which the localized changes contribute to overall bone strength is not clear. It has been suggested that nonuniform inelastic deformation over large surface areas increased energy dissipation over a uniform material.(63) The heterogeneity of the localized elastic modulus distribution over the trabecular bone surface may inhibit crack propagation or failure in the bone. However, this concept requires additional experiments.

Trabecular bone strength measured by compression of the lumbar trabeculae found that, after 60 days of either antiresorptive agents or PTH, the bone strength had all increased to the sham level. Antiresorptive agents can rapidly improve bone strength through reduction in remodeling and increasing bone mineralization.(15,20,29) However, PTH treatment may rapidly increase bone strength by changing the trabecular bone microarchitecture including trabecular thickness and connectivity. By day 180, PTH treatment lumbar vertebral compression strength was greater than that in the sham-treated animals. The additional gain in bone strength with prolonged PTH treatment may be that this additional treatment time allowed the osteoid to fully mineralize the bone matrix. Prolonged PTH treatment also improved cortical bone strength, which may be the result of a combination of Haversian remodeling cycle completion and increased cortical bone thickness resulting from endocortical and periosteal bone formation. Recently, 20-wk-old estrogen-replete mice treated for 7 wk had gains in vertebral bone mass between 10% and 13% with alendronate and 14% and 33% with PTH. The combination of the two treatments in both daily and cyclic regimens may be more beneficial than either treatment alone in improving bone quality.(64)

In summary, treatment of estrogen-deficient aged rats with either antiresorptive agents or PTH rapidly improved many aspects of bone quality including microarchitecture, bone mineralization, turnover, and bone strength. However, prolonged treatment for 180 days with PTH resulted in additional gains in bone quality and bone strength suggesting that the maximal gains in bone strength in cortical and trabecular bone sites may require a longer duration of PTH treatment. Other studies that evaluate the role of dose-response of these agents on bone quality will help to further refine our observations and translate this preclinical observation to practice.
